# Notes and News

**Published:** 1896-10-24

**Authors:** 


### Notes and News.

(Collected and Communicated.)

A new wing has been added to the St. Francis Hospital, Trenton, in the United States. Extra accommodation for 45 patients is provided by this means. The cost of the building was 34,000 dols.

The Duchess of Teck opened the New Brentford Union Infirmary last week. The building is on the corridor plan, with pavilions. It will accommodate 264 patients. The cost is estimated at about £40,000.

The building fund of the Newcastle Infirmary, now amounts to £50,000, which includes a legacy of £5,000 from Dr. Heath. It is proposed to lay the foundation-stone of the new infirmary on Coronation Day in 1897.

Mr. G. W. Gostang has been appointed house surgeon at the County Hospital, York. Mr. Gostang, who formerly held the post of assistant house surgeon, has succeeded Mr. Hodgson, who resigned his post, which he held for three years.

As at many other places, an agitation is going on at Colchester against the abuse of the out-patient department of the hospital. From the correspondence published in the local papers, the "letter system" seems to be at the bottom of much of the difficulty.

The outbreak of Beri-Beri at the Richmond Asylum, Dublin, shows no sign of abatement. Upwards of seventy persons have been attacked, amongst whom were several nurses. Dr. Conolley Norman proposes to deal with the subject during the approaching session at the Academy of Medicine.

It is proposed, by way of meeting the difficulty of securing a site for a fever hospital for Chiswick, to establish two floating hospitals in the Thames. The proposal is that these hospitals—one for fever cases, and the other for small-pox—should be moored alongside of the floating hospitals of the Metropolitan Asylums Board at Dartford, and that patients should be conveyed from Chiswick by steam launch.

At Bristol the Joint Hospitals Committee will hold a series of amateur performances at the Prince's Theatre on behalf of the medical charities of the city.

It has been proposed to establish a hospital for tuberculosis in Philadelphia, under the supervision of the Board of Health, on the lines of the Municipal Hospital for Contagious Diseases.

Dr. Lane Notter will deliver his inaugural address at the first meeting of the Epidemiological Society on November 20th, at eight p.m. "The Epidemic Prevalence of Infective Disease in the Tropics" will form the topic.

We learn that two private hospitals have been established in St. Louis, in America, by associations the members of which are entitled to free treatment. This is a novel development of the home hospital system, whereby it would appear that the ailing will benefit at the expense of the healthy.

The foundation-stone of the new convalescent home in connection with the Metropolitan Hospital was laid last week at Cranbrook by Mrs. F. S. Cornwallis. The site, which is suitable and picturesque, was given by Mr. Cornwallis, whilst Mr. Passmore Edwards has contributed £3,000 towards the cost of the building.

A good deal has been said lately about the unsatisfactory water supply at Eastbourne. Public opinion in Eastbourne is in favour of the acquisition of the waterworks by the Corporation. Certainly this will throw the important responsibility upon a body directly interested in placing the town in a favourable position, and benefits should result. In the meantime we learn from the *Eastbourne Gazette* that a new supply of drinking water has been added to the resources of the place capable of supplying each resident with ten gallons of water daily. The water is described as pure spring water, and therefore it is to be hoped that visitors to Eastbourne will no longer have to face the very unpleasant flavour which was met with in the water previously used.

Cheltenham has this year gone through an experience in regard to its water supply which well illustrates one of the risks by which artificial arrangements for the storing of water are liable to be upset. In March the water from the principal reservoir was found to be turbid and to give off an offensive odour, especially when heated, and a little later the water in the reservoir itself was found to be of a red colour, although that in the upper settling-pool was of the same green tint which usually characterised it. The red colour and turbidity were not entirely removed by filtration, but the filters quickly became clogged, and from the heap of sand scraped from the filter-bed (according to the description furnished by Dr. Garrett to *Public Health*) there drained a thick, dark red, semi-fluid juice, which had a strong and offensive odour, like a farmyard when the manure of a year is being removed, and when dried by the sun it became almost black, and cracked and curled like dried blood. All this trouble seems to have been caused by the growth of a certain micrococcus, analogous, if not identical, with one described by Professor Zopf under the name of Crenothrix polyspora, which was found to be swarming in the water in enormous multitudes. It would be interesting to see whether the introduction of some fish into these reservoirs would not prevent a recurrence of this difficulty.

